# Investigation of the effects of astaxanthin in experimental polycystic ovary syndrome (PCOS) in rats

**DOI:** 10.22038/IJBMS.2023.69984.15223

**Published:** 2023

**Authors:** Erdem Toktay, Jale Selli, Muhammet Ali Gurbuz, Raziye Alaca

**Affiliations:** 1 Department of Histology and Embryology, Faculty of Medicine, Kafkas University, Kars, Turkey; 2 Department of Histology and Embryology, Faculty of Medicine, Alanya Alaaddin Keykubat University, Antalya, Turkey; 3 Department of Histology and Embryology, Faculty of Medicine, Ataturk University, Erzurum, Turkey; 4 Philosophy Doctor Degree, Department of Histology and Embryology, Erzurum City Hospital, Erurum, Turkey

**Keywords:** Astaxanthin, Metformin, Ovary, Oxidative stress, Polycystic ovary syndrome

## Abstract

**Objective(s):**

The aim of this study was to investigate the effect of Astaxanthin (ASX) on ovaries in letrozole-induced polycystic ovary syndrome (PCOS) model in female rats by histopathological, immunohistochemical and biochemical techniques.

**Materials and Methods:**

Seventy two Sprague-Dawley female rats with an average weight of 200-250 gr and 10-12 weeks old were randomly divided into 9 groups. PCOS model was applied to all groups except healthy group. In the study, low (10 mg / kg) moderate (20 mg / kg) and high (40 mg / kg) doses of ASX were given to the experimental animals in the PCOS-induced groups for 7 days. At the end of the experiment, ovarian tissues were evaluated histopathologically, immunohistochemically, and biochemically.

**Results:**

When the histopathological findings were examined, many cystic follicles, apoptotic and necrotic cells were found in the follicles in the PCOS group. In addition, significant decrease in apoptotic and necrotic cells were observed in PCOS+MET+ASX and PCOS+ASX groups. In immunohistochemical staining findings, while TNF-α NF-κB and IL-6 expression levels showed significant increase in PCOS group, these expression levels were decreased in PCOS+MET+ASX and PCOS+ASX groups. In the biochemical evaluations, while MDA were increased, SOD were decreased in the PCOS group. MDA level were decreased while SOD levels were increased in the PCOS+MET+ASX and PCOS+ASX groups.

**Conclusion:**

In addition to the formation of insulin resistance in the tissue, severe oxidative stress damage occurs in ovarian tissue during PCOS. Metformin improved PCOS by correcting insulin resistance. In this period, the administration of ASX with Metformin protected the ovary from oxidative stress damage

## Introduction

Polycystic ovary syndrome (PCOS) is an endocrine disease that causes disruptions in the ovarian cycle that occurs every month from puberty to menopause in women. Irregular menstrual cycles, weight gain, and infertility are common in women with PCOS (1). In the clinic, individuals with both oligomenorrhea (abnormal menstruation), high androgen levels, and ovarian cyst findings are diagnosed with PCOS (2).

Under normal physiological conditions, androgen hormone synthesized from theca cells is converted to estrogen via aromatase enzyme in granulosa cells. However, a deficiency in aromatase enzyme activity triggers an increase in androgen hormones from the ovary (3). High androgen stops the development of antral follicles and leads to the development of many cystic follicles. Experimental research studies shows that administration of the aromatase inhibitor letrozole lead to PCOS in rats (4). Excessive androgen production and conversion of the excess androgens to estrogenic hormones in peripheral adipose tissue results in increased estrogen levels (5). The excess estrogen levels trigger to a decrease in FSH levels that are expected to increase during the ovulation period from the pituitary gland, and anovulation occurs (6). This event, which takes place during successive cycles, leads to the development of many cystic follicles and infertility due to PCOS.

Hormonal axis disorders are not the only problem affecting female infertility in PCOS. It has been reported that excessive free radical production in the ovary in PCOS has adverse effects on female fertility (7). The increase in oxidative stress in PCOS, stimulates apoptosis by affecting the follicle and its microenvironment (8). Oxidative stress affects both developing and immature follicles, and can cause decreased oocyte quality and infertility (9). In studies, findings such as the high number of apoptotic granulosa cells and increased oxidative stress markers in patients with PCOS clearly show the PCOS-oxidative stress-infertility relationship (8, 10). 

Correction of hormonal axis disorders and prevention of oxidative damage in PCOS treatment can prevent infertility (11). Effective hormonal agents and metformin that eliminates the insulin resistance frequently seen in PCOS are clinically used in the treatment (12, 13). Although these protocols are effective in the treatment of PCOS, oxidative stress usually ignored.

Astaxanthin (ASX), an anti-oxidant and antineoplastic agent, is a lipophilic red-orange carotenoid (14). It is abundant in seafood (crustaceans, fish, etc.), algae, and various plants (15). Today, astaxanthin is used as an additive and colorant in various foods in developed countries such as the USA, Japan, South Korea, and Sweden (16). ASX is the strongest anti-oxidant in the group of carotenoids (17). In addition, ASX consist in anti-oxidant 65 times more than vitamin C, 54 times more than β-carotene, and 14 times more than vitamin E; It has been shown to be 10 times more potent than zeaxanthin, lutein, and canthaxanthin, and 100 times more potent than alpha-tocopherol (18). ASX has a unique structure that can interact with both sides of the cell membrane due to its lipophilic feature (19). Thanks to this feature, it has been shown that it protects the cell membrane structure from oxidative damage and inhibits the formation of lipid peroxidation products (20). Another feature of ASX has plays a role in glucose metabolism and has an effect on the adjustment of insulin resistance (21). This feature suggests that it may play a role similar to metformin as a biomolecule in diseases related to glucose metabolism.

Briefly, ASX may act as both a protective agent against oxidative stress and a therapeutic agent by adjustment insulin resistance in PCOS. For this purpose, in our study, the effect of ASX in a PCOS experimental animal model induced with an aromatase inhibitor in rats was investigated by histopathological, immunohistochemical, and biochemical methods.

## Materials and Methods

***Ethics committee approval***

Our study is approved with the decision no 131 of the 6.meeting of XXX University Animal Experiments Local Ethics Committee dated 22.05.2018.

***Supply of experimental animals and shelter opportunities***

A total of 72 Sprague-Dawley female rats with an average weight of 200-250 grams to be used in our study were obtained from the laboratory of xxx University Experimental Research and Application Center (ATADEM). During the experiment, the experimental animals were given sufficient (ad libitum) water and pellet feed. The provided animals were housed and fed in groups at normal room temperature (24 °C) in the laboratory.

***Experiment model and groups***

To generate PCOS in experimental animals, 1 mg/kg letrozole (Merck, Germany) was dissolved in 1 ml of carboxymethyl cellulose (CMC) and given orally for 21 days to eight groups except the healthy group. From the 21^st^ day onwards, daily vaginal smears were taken from the animals in order to determine whether the PCOS model was formed, and rats with repeated oestrus cycles were evaluated as positive for the PCOS model and were selected for the experiment. Selected PCOS positive animals were administered 20 mg/kg Metformin and ASX 10, 20 and 40 mg/kg doses for 7 days as shown in Table 1. All of the applications for the groups were given in Table 1.

***Surgical procedures***

At the end of the experiment, all animals were anesthetized by intraperitoneal injection with a mixture of 100 mg/kg ketamine (Ketalar, Pfizer) and 15 mg/kg Xylazine (Xylanzinbio, Bioveta). The muscle stretch reflex of the animals was checked and their unconscious state was confirmed. Then, two incisions of 3 cm were made transverse from the diaphragm line and sagittal from the median line of the abdominal region, and the abdomen was opened. The intestines were taken outwards and the double horn structure of the uterus was observed. By following these horns, the ovaries were reached and carefully removed. 

While some of the removed ovarian tissues were taken to -80 degrees for the biochemical process, some were placed in a 10% formalin solution for histopathological examination.

***Histolopathological analysis***

*Tissue processing and preparation of sections*

At the end of the surgical procedure, ovarian tissue samples in formalin solution were placed in plastic tissue processing cassettes and labeled after 48 hr of fixation. After washing under running water for 2 hr, tissue samples were placed in a semi-automatic tissue tracking device (Leica TP 1050) and the tissue tracking process was started. With the protocol of the relevant device, respectively; Dehydration was performed by passing through a series of 50% (2 hr), 70% (1 hr), 80% (1 hr), 96% (1 hr), and 99% (1 hr) alcohol. Subsequently, tissue transparency was performed by passing it through three series of xylene (3x15 min). Finally, the tissues were passed through soft and hard paraffin series (46-48^o ^C- 1 hr and 56- 58^o ^C - 1 hr) and the follow-up process was completed.

At the end of the tissue processing, the plastic cassettes were opened and the tissue samples were fixed in the metal blocking mold in accordance with the section plane. The metal block containers were filled with molten paraffin at 56-58 ^o^C and allowed to freeze at room temperature, and the blocking process was completed. The obtained blocks were placed in a semi-automatic microtome device and sequential serial sections of 5 micrometer thickness were taken from each block. Obtained serial sections from the microtome were taken on polylysine and positively charged slides with the help of mild water water for histopathological and immunohistochemical examination.

*Staining pre-process*

Ovarian tissue samples taken on a poly-lysine coated slide were kept in an oven at 60^o^C for 20 min in order to remove the paraffin and fix the tissue on the slide. Paraffin was completely take away by passing it through the xylene series three times for five minutes. Slides were kept in decreasing alcohol series (99%, 96%, 80%, 70%, 50%) for two minutes to move the xylene away from the tissue and closer to the water. Washing was done in running water for 5 min in order to remove the alcohol.

*Hematoxylin and eosin staining process*

Nucleus staining was performed with Harris hematoxylin (Merck, Germany) dye for three minutes following the staining pre-treatment. At the end of the dyeing process, washing was done in running water for five minutes to remove excessive dye. Then, slides were waited in Eosin Y (Merck, Germany) solution for 2 min for counterstaining. In order to remove excess dye, the slides were slowly dipped in 96% alcohol five times and removed, and then waited in 100% alcohol for two minutes and in three series of xylol for two minutes. Finally, tissue surfaces were covered with coverslip using entellan bonding balm.

*Immunohistochemical staining process*

For immunohistochemical analysis, 5 μm sections of paraffin embedded tissues were taken on positively charged glass slides with a microtome, and immunohistochemical staining was performed by following the procedures listed below. Slides were placed on automated immunohistochemistry stainer (Ventana BENCHMARK GX). After staining, the slides removed from the device were cleaned from the oily surface by dipping them in soapy water several times. Then, Slides was waited for two minutes in 100% alcohol and two minutes in three series of xylol. Finally the tissue surfaces were covered with a coverslip using entellan. Antibodies used in staining, monoclonal NF-κB p65 (Santa Cruz: sc-8008, USA), TNF-α (Santa Cruz: sc-52746, USA) and IL-6 (Santa Cruz: sc-57315, USA) primary antibodies, were used considering the recommended dilution ratio. 

*Microscopic examination and photography*

The preparations prepared at the end of hematoxylin-eosin and immunohistochemical staining were examined and photographed using a computer-assisted Nikon Eclipse E600 (Japan) microscope with camera attachment. The micrographs taken were brought together with Adobe Photoshop CS6 (USA) program for easy comparative analysis.

*Biochemical analysis*

For determination of oxidant and anti-oxidant parameters (MDA and SOD), 100 mg of ovary tissue was weighed for each animal. The tissue was homogenized in phosphate buffered saline solution using homogenizer device. Tissues were centrifuged at high speed and measurements were made from the supernatant with ready-made elisa kits (SunRed, Chine).

*MDA and SOD measurement*

MDA and SOD measurement were performed according to the protocol of the ready-made ELISA kit (SunRed, 96 wells) specially prepared for the rat. Standard solutions were prepared according to the dilutions specified in the kit manual. 50 µl of standard solution was added to the standard wells. 40 µl of sample and 10 µl of anti-E antibody were added to the sample wells. Then, 50 µl of streptavidin-HPR was added to all wells (except for the blind control well). After mixing well with light movements, the well plate was covered with an adhesive tape in such a way that air did not enter and left to incubate at 37 degrees for 60 min. After the incubation, the adhesive tape was removed and the liquids in all the wells were poured, and they were washed 5 times with the help of a washing device (Thermo Scientific Well wash 1x8, Cat. No: 5165000). Fifty µl of solution A and then 50 µl of solution B were added to all wells. Again, the well plate surface was covered with adhesive tape and incubated at 37 degrees for 10 min. At the end of the incubation, 50 µl of stop solution was added to each well and the reaction was stopped. Measurement was made at 450 nm wavelength within 15 min after the addition of the stop solution.

***Statistical analysis***

The data of our study were statistically evaluated with the IBM 20.00 SPSS (USA) program. Biochemical MDA and SOD analyzes were evaluated according to Tukey test. Accordingly, significance value was accepted for *P*<0.05.

In our study, semi-quantitative scoring was performed in immunohistochemical evaluation. Since this method is widely preferred for understanding immunohistochemical analyses, we also used this method in our research. According to this; At least five areas were evaluated for each ovarian slide, and scoring was done considering the number of cells stained and the staining intensity (22).

According to the scoring, if there is little or no immune reactivity – (0%), if immune positivity is mild + (0–30%), if it is moderate ++ (30–60%), and if severe +++ (60–100%) (23).

## Results

***Histopathological findings***

*Hematoxylin and eosin staining findings*

**Figure 1:**
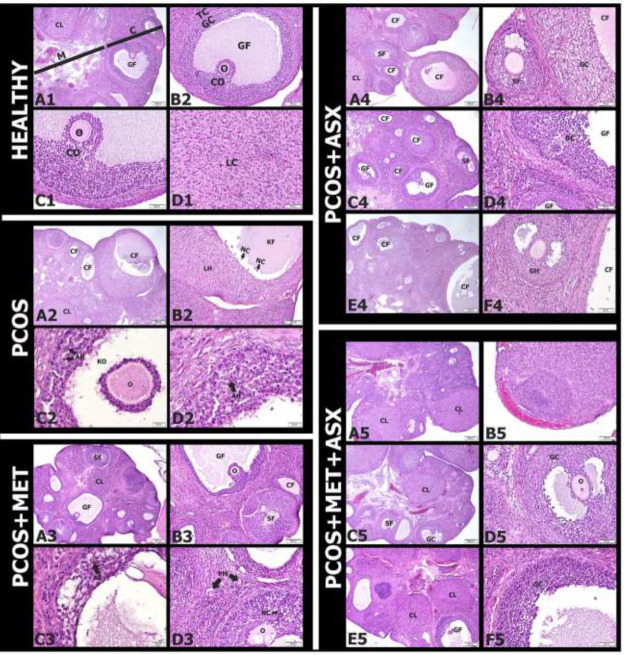
Histopathological findings of effects of astaxhantin on polycystic ovary syndrome in rats; Healthy group (A1-D1), PCOS group (A2-D2), PCOS+MET group (A3-D3), PCOS+ASX10 group (A4-B4), PCOS+ASX20 group (C4-C4), PCOS+ASX40 group (E4-F4), PCOS+MET+ASX10 group (A5-B5), PCOS+MET+ASX20 group (C5-C5), PCOS+MET+ASX40 group (E5-F5)

Cortex and medulla were clearly distinguished in the histopathological examination of the images of the healthy group. Numerous normal-appearing blood vessels were observed in the medulla. Follicles and corpus luteum structures of different sizes and stages were observed in the cortex. When the detailed structure of the Graf follicle was examined, normal-looking granulosa cells, theca cells, and oocytes were distinguished. The cumulus oophorus structure connecting the oocyte to the granulosa cells was observed in the Graf follicle. Normal-looking luteal cells were observed in the corpus luteum (Figure 1. A1-D1).

In the PCOS group, cystic follicles of different sizes and many damaged follicles at the developmental stage were found in the cortex. In particular it was noteworthy that some of the cystic follicle structures changed into the corpus luteum. In granulosa cells of cistic follicles were determined as necrotic appearance cells (Figure 1.A2-B2). It was observed that the cumulus oophorus structure was lost due to the damage in the Graafian follicle. The apoptotic cells were also seen in the granulosa cells of this follicle (Figure 1. A2-D2).

In the PCOS+ MET group, small cystic follicles were rarely observed in the cortex. Secondary and graph follicles in many different stages were observed. The corpus luteum structure consisting of granulosa and luteal cells was observed in the cortex. Necrotic cells were found in cystic follicles, apoptotic cells were rarely observed in some secondary follicles in the developmental stage. In addition, zona pellucida integrity were lost and scattered oocyte structures of the follicles in the developmental stage (Figure 1. A3-D3).

In the PCOS+ ASX10 group, many cystic follicles were observed in the cortex. Necrotic cells were found in granulosa cells around the cystic follicle and the secondary follicle (Figure 1. A4-B4).In the PCOS+ ASX20 group, many cystic follicles were observed in the cortex region. However, the granulosa cells of the secondary follicle and the Graf follicle were normal. Apoptotic and necrotic cells were not detected (Figure 1. C4-D4). In the PCOS+ ASX40 group, many cystic follicles were observed in the cortex, but the apoptotic cells observed in the PCOS group were not observed in the follicle cells at different stages of development. The zona pellucida structure observed around the oocyte was normal. It surrounded the oocyte as a relatively thick extracellular coat (Figure 1. E4-F4).

In the PCOS+ MET+ ASX10 group, no cystic follicle was observed in the cortex. Secondary and graafian follicles of different sizes were seen in cortex. Deflated cystic follicle structures filled with erythrocyte cells under the tunica albuginea was observed (Figure 1. A5-B5). In the PCOS+ MET+ ASX20 group, no cystic follicle was observed in the cortex. In addition, many normal-looking follicles were found in the cortex. The structure of the zona pellucida surrounding the oocyte in the secondary follicle was similar to the healthy group. It was not overlooked that necrotic and apoptotic cells were not observed in the granulosa cells of this follicle (Figure 1. C5-D5). In the PCOS+ MET+ ASX40 group, while no cystic follicles were observed in the cortex, secondary and Graafian follicles at different stages of development were observed. Apoptotic and necrotic cells were not observed in healthy-looking primary, secondary and Graafian follicle granulosa cells (Figure 1. E5-F5).

***Immunohistochemical findings***

**Figure 2:**
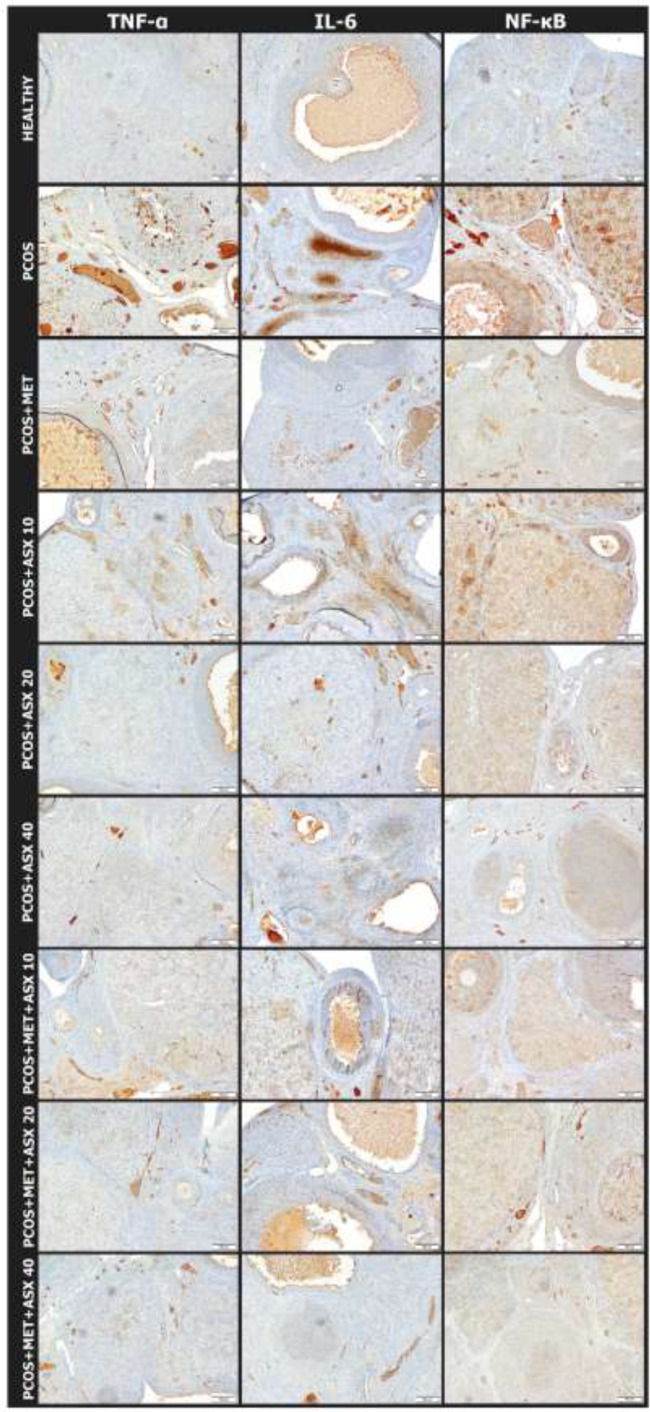
TNF-α, IL-6 and NF-kβ antibody immunohistochemical staining findings of effects of astaxhantin on polycystic ovary syndrome in rats

In TNF-α antibody immunohistochemical staining; mild (+) immune positivity was observed in PCOS+MET, PCOS+ASX10, PCOS+ASX20, PCOS+ASX40, PCOS+MET+ASX10, and PCOS+MET+ASX20 groups. Severe (+++) immune positivity was observed in the PCOS group, while Immune negativity (-) was observed healthy and PCOS+MET+ ASX40 groups (Figure 2).

In IL-6 antibody immunohistochemical staining; mild (+) immune positivity was observed in PCOS+MET, PCOS+ASX10, PCOS+ASX20, PCOS+ASX40, PCOS+MET+ASX10, and PCOS+MET+ASX20 groups. Severe (+++) immune positivity was observed in the PCOS group. Immune negativity (-) was observed in the healthy and PCOS+MET+ASX40 groups as well (Figure 2).

In NF-kβ antibody immunohistochemical staining; mild (+) in PCOS+MET, PCOS+ASX10, PCOS+MET+ASX10, PCOS+MET+ASX20 and PCOS+MET+ASX40 moderate (++) in PCOS+ASX20 group, severe (+++) in PCOS group immune positivity was observed, while immune negativity (-) was observed in the healthy and PCOS+ASX40 groups (Figure 2).

***MDA and SOD levels analysis***

**Figure 3:**
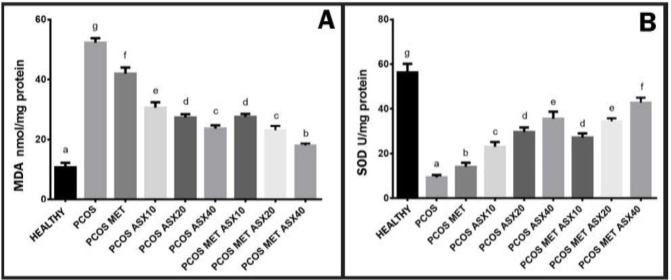
MDA and SOD biochemical findings of effects of astaxhantin on polycystic ovary syndrome in rats

According to evaluation of Malondialdehyde levels; While the MDA level increased dramatically in the PCOS group, a significant decrease was detected in the PCOS+MET group compared to PCOS (*P*<0,05). It was found that increasing doses of ASX produced a significant reduction compared to both the PCOS and PCOS+MET groups. In addition, it was determined that there was a significant decrease in PCOS+MET+ASX groups compared to PCOS+ASX groups (*P*<0.05) (Figure 3A).

According to the evaluation of SOD levels; While it was observed that the SOD level decreased dramatically in the PCOS group compared to the control group (p<0,05) , it was found to be significantly increased in the PCOS+MET group compared to PCOS (*P*<0,05). It was determined that increasing doses of ASX produced a significant increase compared to both the PCOS and PCOS+MET groups. In addition, it was determined that there was a significant increase in PCOS+MET+ASX groups compared to PCOS+ASX groups. (Figure 3B).

**Table 1:** Experimental groups and design to investigate effects of astaxhantin on polycystic ovary syndrome in rats

Groups	Groups Name	Application
1. Groups	HEALTHY	It was kept until the end of the experiment without applying any drug.
2. Groups	PCOS	It was administered 1 mg/kg of Letrozole for 21 days.
3. Groups	PCOS + MET	It was administered 1 mg/kg of Letrozole for 21 days. Then, 20 mg/kg Metformin was administered for 7 days
4. Groups	PCOS+ASX10	It was administered 1 mg/kg dose of Letrozole for 21 days. Then, 10 mg/kg Astaxanthin was administered for 7 days.
5. Groups	PCOS+ASX20	It was administered 1 mg/kg dose of Letrozole for 21 days. Then, 20 mg/kg Astaxanthin was administered for 7 days.
6. Groups	PCOS+ASX40	It was administered 1 mg/kg dose of Letrozole for 21 days. Then, 40 mg/kg Astaxanthin was administered for 7 days.
7. Groups	PCOS+MET+ ASX10	It was administered 1 mg/kg dose of Letrozole for 21 days. Then, 20 mg/kg Metformin and 10 mg/kg Astaxanthin was administered for 7 days.
8. Groups	PCOS+MET+ ASX20	It was administered 1 mg/kg dose of Letrozole for 21 days. Then, 20 mg/kg Metformin and 20 mg/kg Astaxanthin was administered for 7 days.
9. Groups	PCOS+MET+ ASX40	It was administered 1 mg/kg dose of Letrozole for 21 days. Then, 20 mg/kg Metformin and 40 mg/kg Astaxanthin was administered for 7 days.

PCOS: Polycystic ovary syndrome, MET: Metformin and ASX: Astaxhantin

**Table 2:** Immunohistochemical staining results scoring table of effects of astaxhantin on polycystic ovary syndrome in rats

GROUPS	TNF α	IL-6	NF-kβ
HEALTHY	**-**	**-**	**-**
PCOS	**+++**	**+++**	**+++**
PCOS+MET	**+**	**+**	**+**
PCOS+ASX10	**+**	**+**	**+**
PCOS+ASX20	**+**	**+**	**++**
PCOS+ASX40	**+**	**+**	**-**
PCOS+MET+ASX10	**+**	**+**	**+**
PCOS+MET+ASX20	**+**	**+**	**+**
PCOS+MET+ASX40	**-**	**-**	**+**

Healthy, PCOS: Polycystic ovary syndrome, MET: Metformin, ASX: Astaxhantin, TNF-α: Tumor Necrosis Faktor alfa, IL-6: Interleukin 6 and NF-κB: Nuclear factor kappa B

C: Cortex, M: Medulla, PM: Primary follicle, SF: Secondary follicle, GF: Graafian follicle, KL: Corpus luteum, GC: Granulosa cells, TC: Theca cells, CO: Cumulus oophorus, O: Oocyte, LC: Luteal Cells, CF: Cystic follicle, AC: Apoptotic Cells, NC: Necrotic cells, PCOS: Polycystic ovary syndrome, MET: Metformin and ASX: Astaxhantin

Magnification rate: 10x10-Brown staining cells indicates cells that stained immunohistochemically

PCOS: Polycystic ovary syndrome, MET: Metformin, ASX: Astaxhantin, TNF-α: Tumor necros faktor alfa, IL-6: Interleukin 6 and NF-κB: Nuclear factor kappa B

PCOS: Polycystic ovary syndrome, MET: Metformin, ASX: Astaxhantin, MDA: Malondialdehyde and SOD: Superoxide dismutase

## Discussion

PCOS is a clinical problem of unknown cause (24). It is thought that environmental, epigenetic, and hormonal factors play an important role in the pathogenicity of the disease (5). As a result of exposure to androgens, PCOS is characterized by abnormal follicle development, disorders in estrogen and progesterone hormone secretion, and abnormal menstrual cycle (25, 26). In addition, high levels of oxidative stress occur in the ovary during PCOS. Reactive oxygen species (ROS) lead to the deterioration of the anti-oxidant defense system (27-30). The ROS is a threat to many mature and immature follicles in the ovary. It has similarly been shown that oxidative stress indirectly affects the reproductive organs in the form of endometriosis and unexplained infertility. (27-29). 

In this study, PCOS model was created with letrozole, which an inhibitor of the aromatase enzyme that breaks down estrogen hormone (31, 32). The effect of ASX on PCOS, a powerful anti-oxidant, was investigated by using it alone or in combination with metformin. 

The presence of cystic follicles observed in the PCOS group in the microscopic examination, our experimental animal model was successfully formed. The presence of apoptotic and necrotic cells in the follicles we observed in the PCOS group proves the presence of oxidative stress. There are many studies supporting these findings (33, 34). The almost absence of cystic follicles in the PCOS+ MET group explains the use of this drug in routine treatment (35, 36). However, the presence of apoptotic and necrotic cells observed in the follicles reveals that metformin is insufficient in eliminating the oxidative stress. In the increasing dose groups of PCOS in which Metformin was not administered but Astaxanthin was administered, it was observed that the cystic follicles that emerged due to PCOS continued to exist. However, it has been revealed that the application of astaxanthin plays a protective role in other developing follicles. In addition to the disappearance of cystic follicles in the metformin-administered and astaxanthin increasing dose groups of PCOS, other follicles that developed significantly were preserved. These results are similar to the findings of studies in the literature. We observed this protective effect of astaxanthin against oxidative damage in our previous ovarian ischemia-reperfusion injury (23).

One of the most obvious indicators of cell and tissue damage is the presence of oxidative stress. For this purpose, in the biochemical findings of our study, MDA levels, which caused by oxidative stress, especially in the cell wall, were measured. It shows that the significant increase seen in the PCOS group is compatible with the literature. However, the significant decrease in MDA levels in the metformin-administered and non-metformin-administered astaxanthin groups of PCOS indicates that increasing doses of ASX have a positive effect on oxidative stress. The review by Mursi *et al*., showing the relationship between oxidative stress and PCOS, supports our findings (37).

The presence of enzymes such as superoxide dismutase plays an important role in the elimination of oxidants that occur in the organism (38). In our study, we had the chance to obtain general information about intra-tissue anti-oxidant systems by analyzing SOD enzyme levels. The significant dramatic decrease observed especially in the PCOS group revealed that the anti-oxidant system within the tissue shifted towards damage. The fact that astaxanthin administration increased SOD levels in the groups was found to be positive regarding the removal of tissue damage. The study of Belendra *et al*. showing that Astaxanthin increases SOD levels in Alzheimer’s disease supports our findings (39). In another astaxanthin study, SOD levels were increased in the treatment groups (40).

The continuous oxidative stress in the tissue leads to the activation of other systems that trigger the elimination of damaged cells. TNF-α, which plays a role in this process, known as inflammation, is one of the biomediators that occur in the case of oxidative stress. (41). This cytokine produced by macrophage cells in the tissue interacts with the surface receptors of damaged cells and drives the cell to apoptosis, known as the death cascade. In this respect, the increased immune positivity level in our study, especially in the PCOS group, shows the degree of tissue damage. In addition, the decrease in immune response in ASX applied groups was considered as a remarkable finding. The fact that Wu *et al*. investigated the relationship between abnormal HPS70 levels and PCOS and showed that TNF-alpha levels were high in the PCOS group, supports our findings (42).

IL-6 is one of the most important inflammatory mediators. It plays an initiating role in the tissue, especially for the events that will occur later (43). The oxidative damage that occurs in PCOS leads to the deterioration of lipid structures and the emergence of many apoptotic and necrotic cells. IL-6 plays a major role in directing phagocytic cells to eliminate these cells. (43-46). In the PCOS group of our study, especially the increased degree of immunopositivity gives us information about the damage in the tissue with this aspect. As a matter of fact, decreased IL-6 immunopositivity levels in parallel with the elimination of superoxides in the ASX treatment groups indicate that tissue damage is reduced. 

NF-kB is a transcription factor that activates pathogens and inflammatory mediators. NF-kB transcription factor is required for mRNA synthesis of mediators such as IL-6 and TNF alpha inside the cell. The high level of expression in the PCOS group in our study proves the PCOS study of Gonzalez *et al*. (47). On the other hand, a dose-related decrease in NF-kB levels was observed in the treatment groups. This NF-kB finding, which supported our findings, were consistent with TNF alpha and IL-6 levels in our study. 

In addition to all this information, the clinical study showing that ASX alleviates oxidative stress in PCOS patients in a newly published (48), has made the findings of our experimental study more striking. 

## Conclusion

In addition to the formation of insulin resistance in the tissue, severe oxidative stress damage occurs in ovarian tissue during PCOS. Metformin improved PCOS by correcting insulin resistance. Routine metformin therapy plays a key role in the reduction of cystic follicles in PCOS. However, the use of metformin alone is not sufficient to relieve oxidative stress. In this period, the administration of ASX with Metformin protected the ovary from oxidative stress damage. Briefly; Individuals diagnosed with PCOS need to take a strong anti-oxidant such as Astaxanthin (40 mg/kg) to protect the ovarian tissue from oxidative stress damage, whether they receive treatment or not.

## Authors’ Contributions

E T, J S, and MA G designed the experiments; MA G and R A performed experiments and collected data; E T, J S and MA G discussed the results and strategy; E T and J S Supervised, directed and managed the study E T, J S, R A and MA G Final approved of the version to be published (the names of all authors must be listed).

## Conflicts of Interest

All authors declare no conflict of interest.
